# Association between prior-night sleep and next-day fatigue in older adults: a daily diary study

**DOI:** 10.1186/s12877-023-04539-0

**Published:** 2023-12-07

**Authors:** Takeshi Nakagawa, Saori Yasumoto, Mai Kabayama, Ken’ichi Matsuda, Yasuyuki Gondo, Kei Kamide, Kazunori Ikebe

**Affiliations:** 1 Research Institute, National Center for Geriatrics and Gerontology, 7-430, 474-8511 Morioka, Obu, Aichi Japan; 2 Graduate School of Human Sciences, Osaka University, Osaka, Japan; 3 Graduate School of Medicine, Osaka University, Osaka, Japan; 4 Graduate School of Dentistry, Osaka University, Osaka, Japan

**Keywords:** Emotion, Daily diary, Fatigue, Sleep duration, Sleep quality, Within-person

## Abstract

**Background:**

Fatigue is known as an element of frailty. Sleep problems (e.g., short sleep duration and low sleep quality) can increase fatigue, but the day-to-day relationship between sleep and fatigue has not been studied well in older adults. Using a daily diary method, this study examined the within- and between-person associations between sleep and fatigue in older adults.

**Methods:**

The study recruited 56 Japanese community dwellers (age: 82–86 years; female: 37.5%). Participants responded to a daily diary questionnaire at the end of each day. Over seven days, time in bed and satisfaction were measured after waking up, whereas fatigue was assessed before going to bed. We included person-level covariates (demographic factors, and physical and mental health) and day-level covariates (time in study, and positive and negative emotions). Multilevel models were estimated to examine within- and between-person associations.

**Results:**

At the within-person level, on days following short and long time in bed and days following low levels of sleep satisfaction, individuals felt higher levels of fatigue compared with usual days. At the between-person level, no statistically significant differences in fatigue were observed between individuals with long and short time in bed.

**Conclusions:**

The findings suggest that prior-day sleep is associated with next-day fatigue in older adults. Long and short sleep duration and low sleep quality can lead to fatigue. Considering that sleep is a modifiable health behavior, appropriate management of sleep behavior may reduce fatigue.

**Supplementary Information:**

The online version contains supplementary material available at 10.1186/s12877-023-04539-0.

## Background

Fatigue has been considered one of the core elements of frailty that indicate the status of physiological vulnerability to stressors associated with aging [1, 2]. The majority of studies have operationalized and measured fatigue as a “general feeling of tiredness” [3] and revealed the association between fatigue and adverse health outcomes, such as disability and mortality [4–7]. Thus, accumulative evidence indicates that fatigue represents one of the indicators of decreased physiological flexibility associated with aging [8, 9]. In fact, several studies have reported the high prevalence of fatigue in older adults [10–12]. The mechanisms underlying fatigue remain unclear, but researchers [8, 13] have proposed biological (e.g., inflammatory mediators) and psychological factors (e.g., mood disorders). Exploring possible factors involved in the occurrence of fatigue may provide suggestions for the formulation of approaches to improve interventions for fatigue.

Prior research has focused on sleep behavior as a potentially relevant factor in fatigue, given that sleep problems become more prevalent in old age and can lead to adverse health outcomes [14]. Cross-sectional studies incorporating older patients with cancer or osteoarthritis reported that sleep problems (i.e., low sleep quality and sleep disturbance) were associated with fatigue [15, 16]. A longitudinal study on older patients has demonstrated that the improvement of insomnia predicts reduced fatigue [17]. Moreover, longitudinal studies on community-dwelling older adults revealed that sleep problems (i.e., sleep disturbances, such as difficulty in falling asleep, difficulty in staying asleep, and short sleep duration) predicted fatigue [18, 19]. These studies have suggested that sleep behavior is associated with fatigue in older adults. However, several questions warrant further inquiry.

First, it remains unclear whether the existing findings obtained from between-person comparisons can be generalized to within-person associations [20, 21]. Specifically, the majority of studies have compared people with and without sleep problems and have indicated that people with sleep problems were more likely to experience fatigue [12, 18, 19]. In daily life, however, sleep behavior fluctuates on a daily basis. In other words, one may or may not sleep well for several days. To address the limitations of previous studies that have adopted a between-person comparison, recent studies utilize a within-person comparison that compares days with and without sleep problems. For example, a few studies examined the day-to-day relationship between sleep and fatigue in middle-aged adults [22, 23]. DePasquale and colleagues [23] asked middle-aged female workers via telephone interviews for eight evenings to rate sleep using a part of the Pittsburgh Sleep Quality Index (PSQI) [24] and fatigue using a single item. Harris and colleagues [22] asked middle-aged adults with and without sleep disorders via daily diaries for two weeks to rate sleep using Consensus Sleep Diary [25] every morning and fatigue using a visual analog scale every evening. Despite these methodological differences (e.g., data collection procedures and measures), these studies consistently reported that short sleep duration and low sleep quality were associated with more fatigue at the within-person level but that the relationship was observed for sleep quality, but not sleep duration, at the between-person level. Specifically, on days following a short sleep duration and low sleep quality, individuals felt more fatigue compared with typical days [22, 23]. In contrast, when comparing people with long and short sleep duration, no interindividual difference was found in the levels of fatigue. The previous findings highlight the importance of distinguishing within- and between-person associations in investigating the relationship between sleep and fatigue.

Second, whereas the abovementioned studies indicate that sleep problems increase fatigue in middle-aged adults [22, 23], the daily association between sleep and fatigue has been less studied in older adults. A handful of studies suggest long sleep duration may also increase fatigue in older adults. At the between-person level, one study [26] conducted a cross-sectional study and suggested that middle-aged and older adults with short and long sleep duration reported higher levels of fatigue than those with intermediate sleep duration. In the earlier study, sleep was objectively measured using wrist actigraphy, and fatigue was assessed using multiple items, including the Pittsburgh Fatigability Scale [27]. At the within-person level, another study used experience sampling methods and examined associations between sleep and affect balance, indexed as the difference between positive and negative emotions, in a lifespan sample [28]. Sleep duration was assessed using a single-item scale. Although fatigue was not directly measured, the previous study revealed that affect balance in the mornings deteriorated not only on days following short sleep duration but also on days following long sleep duration. Wrzus and colleagues [28] further found that such a curvilinear association between sleep duration and affect balance was more evident in older adults than younger adults. In sum, although prior research has suggested a potential within-person association between short and long sleep duration and fatigue, the day-to-day relationship between sleep and fatigue has not been studied well in older adults.

To fill the gap, this study used a daily diary method and examined the relationship between sleep and fatigue in older adults by distinguishing between- and within-person levels. We hypothesized that individuals would feel high levels of fatigue on days following short and long sleep duration.

## Methods

### Participants and procedures

We conducted this study between 2014 and 2015 as a substudy within an ongoing longitudinal study on community dwellers [29]. The longitudinal study has been conducted at 3- to 4-year intervals since 2011 in four regions in Japan, namely, western-rural, western-urban, eastern-rural, and eastern-urban regions. This daily diary study was conducted only in the two eastern regions. The participants of the longitudinal study were recruited from residential registries and contacted via postal mail. They were invited to participate in the longitudinal study at a survey venue near their homes and gave their informed consent. The exclusion criterion was having been certified as requiring care level 2 or higher in the public long-term care insurance system, indicating physical and cognitive impairments [30].

In this study, we obtained additional written informed consent from the participants at the survey venue. The participants received compensation of approximately 8 USD, which was installed at approximately 4 USD before and after the diary survey. This study was approved by the research ethics review committee of Behavioral Division, Graduate School of Human Sciences, Osaka University (26–036).

At baseline, the participants responded to a questionnaire that measures person-level variables (e.g., living arrangements). They were further instructed to respond to the daily diary. Over seven days beginning with the next day after the baseline survey, the participants responded to the daily diary that assesses day-level variables (e.g., sleep and fatigue) two times per day, namely, after waking up and before going to bed. The participants returned the daily diary via mail after completing the 7-day diary.

To assess day-to-day fluctuations (i.e., an individual-specific deviation from the person–mean; for details, see the Data preparation section), we selected 56 out of 73 participants who responded to the daily diary for at least 2 days (age: mean [*M*] = 83.23, standard deviation [*SD*] = 1.06, *range*: 82–86 years; female: 37.5%). An earlier study [23] also adopted this procedure. Out of a total of 392 days (56 participants × 7 days), on average, the participants responded 6.88 times (*SD* = 0.69) and 6.98 times (*SD* = 0.13) after waking up and before going to bed, respectively.

To evaluate potential selection bias, 17 respondents, who were excluded from analysis because of nonresponse to the daily diary, were compared with those included in the subsequent analysis. The result indicated that the proportion of women was higher in the excluded respondents (${\chi }^{2}\left(1\right)$ = 3.92, *p* = .048; female: 64.7% for nonrespondents). However, no significant difference was observed in age (*t*(71) = 1.23, *p* = .224; *M* = 83.59, *SD* = 1.00 for nonrespondents).

### Measures

#### Outcome variable

We measured fatigue as the outcome variable every day before going to bed using the one item, “tired,” from the four-item fatigue subscale of the expanded version of the Positive Affect-Negative Affect Schedule (PANAS-X) [31, 32]. The participants were asked about the emotion they felt during a given day, with responses ranging from 1 = “never” to 6 = “very frequently” for the item.

Earlier studies used the subscale of the PANAS-X as an indicator of fatigue [33, 34]. In the present study, the multilevel confirmatory factor analysis showed that the between- and within-person standardized factor loadings were high (0.939 and 0.771) for the item “tired.” However, whereas the between-person factor loadings were moderate to high for the items “sluggish,” “sleepy,” and “drowsy” (0.397, 0.615, and 0.846, respectively), the within-person factor loadings were low (0.315, 0.226, and 0.300, respectively). Additionally, the reliability coefficients were high (*ω* = 0.810) at the between-person level but low (*ω* = 0.378) at the within-person level. Based on these results, we chose the item “tired” as the indicator of fatigue.

#### Predictive variables

Sleep behavior during the prior night was measured as the predictive variable every day after waking up. Following previous studies [35, 36], the participants were instructed to record bedtime and waking time, and the time spent in bed between bedtime and waking time was operationalized as sleep duration. Furthermore, a prior study reported that the quantity and quality of sleep are associated with positive and negative emotions [37]; thus, we also measured sleep satisfaction. Considering earlier studies [38, 39], the participants responded from 1 = “I am not satisfied” to 5 = “I am satisfied” to the question, “How satisfied were you with your sleep last night?”

#### Person-level covariates

Demographic factors, including age, gender (0 = *male*; 1 = *female*), and living arrangements (0 = *living with others*; 1 = *living alone*), were measured as person-level covariates, which can be correlated with sleep and fatigue. In addition, we measured physical and mental health, given that previous studies included physical and mental health as relevant factors that may be associated with sleep and fatigue [18, 19, 26].

Physical health was assessed using instrumental independence, which is a subscale of the Tokyo Metropolitan Institute of Gerontology Index of Competence [40, 41]. Previous studies have used the subscale as an index of instrumental activities of daily living [42–44]. The participants were asked if they could perform five activities (e.g., using public transportation and shopping) using a dichotomous response scale. The summary scores ranged from 0 to 5, with high scores indicating better physical health.

Mental health was measured using the Japanese version of the WHO-Five Well-Being Index (WHO-5-J) [45, 46]. The WHO-5-J consists of five questions and has been used as a screening tool for depression. The participants were asked about their mental health during the past two weeks and gave responses ranging from 0 = “at no time” to 5 = “all of the time” to each question. The summary scores ranged from 0 to 25, with high scores indicating better mental health.

#### Day-level covariates

In this study, time was considered the day-level covariate that may be correlated with sleep and fatigue. The first day of the daily diary was coded as 0, whereas the final day was coded as 6. Thus, one unit of time in study corresponded to 1 day.

Moreover, previous studies [23, 47, 48] suggested that daily emotions were associated with sleep and fatigue. Thus, the current study included positive and negative emotions as day-level covariates. Daily emotions were measured every day before going to bed using PANAS [49, 50]. PANAS consists of a total of 16 items, with eight items representing positive or negative emotions. The participants were asked about the emotions they felt during a given day, with responses ranging from 1 = “never” to 6 = “very frequently” to each item. In the subsequent analysis, the total scores of each subscale of positive and negative emotions were calculated and divided by 8 (i.e., the total number of items per subscale), with scores ranging from 1 to 6.

### Data analysis

#### Data preparation

To distinguish between- and within-person associations, time-varying predictive variables and covariates were grouped into two components: between-person differences and within-person variations. The classification is based on standard procedures for the analysis of intensive longitudinal data [21, 51]. Specifically, a between-person component was defined as the within-person average score of repeatedly measured data nested within individuals. In addition, a within-person component was defined as the deviation of an individual from the within-person average levels at each measurement point. For example, time in bed was converted into two variables, namely, the time-invariant variable of person–mean levels of time in bed (e.g., 8 h) and the time-varying variable of deviation from the person–mean time in bed across seven days (e.g., − 2 and 1 h). Moreover, the centering of person-level predictor variables and covariates at sample means enabled the interpretation of intercepts and estimates in the multilevel model as a prototypical individual.

#### Multilevel models

To examine how sleep and fatigue are associated separately at the between-person and within-person levels, we fitted the multilevel models to data where the 7-day data were nested within individuals.

First, the unconditional means models were specified for the day-level variable to calculate interclass correlation coefficients (ICCs). ICCs refer to the ratio of between-person variance to the total variance, and higher values indicate that the variables of interest are more stable over time.

To evaluate the Level 1 (within-person) and 2 (between-person) effects of sleep on fatigue, the Level 1 model was specified as follows.

Level 1:

fatigue_ti_ = β_0i_ + β_1i_(time in study_ti_) + β_2i_(within-person positive emotions_ti_) + β_3i_(within-person negative emotions_ti_) + β_4i_(within-person time in bed_t−1i_) + β_5i_(within-person time in bed^2^_t − 1i_) + β_6i_(within-person sleep satisfaction_t−1i_) + e_ti_,

where fatigue_ti_ denotes the degree of fatigue of person *i* on day *t* and is specified as a function of β_0i_, which refers to an individual-specific intercept parameter; β_1i_ represents an individual-specific parameter of changes per day; β_2i_ characterizes an individual-specific coupling between fatigue and the within-person component of positive emotions; β_3i_ stands for an individual-specific coupling between fatigue and the within-person component of negative emotions; β_4i_ captures an individual-specific linear association between the within-person component of prior-night time in bed and next-day fatigue; β_5i_ pertains to an individual-specific curvilinear association between the within-person component of prior-night time in bed and next-day fatigue; β_6i_ represents an individual-specific association between the within-person prior-night sleep satisfaction and next-day fatigue; and e_ti_ indicates the first-order autocorrelated residuals.

We note that the time metric centered at the median during the observation period (i.e., 7 days) is added to the Level 1 model following Bolger and Laurenceau [51] to control for time-related changes, which are outside the scope of the study. Additionally, to control for autodependency across days in time-varying variables, the study employed the autoregressive covariance structure.

Next, the Level 2 model specified individual-specific intercepts and associations as follows.

Level 2:

β_0i_ = γ_00_ + γ_01_(age_i_) + γ_02_(gender_i_) + γ_03_(living arrangements_i_) + γ_04_(physical health_i_) + γ_05_(mental health_i_) + γ_06_(positive emotions_i_) + γ_07_(negative emotions_i_) + γ_08_(time in bed_i_) + γ_09_(sleep satisfaction_i_) + u_0i_,

β_1i_ = γ_10_,

β_2i_ = γ_20_,

β_3i_ = γ_30_,

β_4i_ = γ_40_,

β_5i_ = γ_50_,

β_6i_ = γ_60_.

In the Level 2 model, γ denotes person-level intercepts and associations, whereas u is an individual-specific deviation from the person–mean intercept. Among the primary variables in this study, the person-level (between-person) association between fatigue and time in bed is denoted by γ_08_, whereas typical within-person associations are denoted by γ_40_ and γ_50_. In the same manner, the between-person association between fatigue and sleep satisfaction is denoted by γ_09_, whereas the typical within-person association is denoted by γ_60_. Notably, the between-person curvilinear association between fatigue and time in bed was statistically nonsignificant from 0. Thus, we assumed only a linear association. However, the model converged when assuming u_0i_, which represents an individual-specific deviation from the person–mean intercept of fatigue (β_0i_). Moreover, the individual deviation was statistically significant, whereas individual deviation from other parameters (i.e., β_1i_ to β_6i_) was nonsignificant. Thus, we assume that these parameters, except for β_0i_, are invariant across individuals, and the model does not specify their random effects.

As follow-up analyses, we explored the reversed associations to examine whether prior-day fatigue was associated with next-day sleep. Due to the lagged design, the number of observations was reduced: Sleep behavior for the first day was excluded from analyses because fatigue was not measured the day before.

The subsequent statistical analyses were performed using SPSS version 28. Missing data in longitudinal data were treated as missing at random [52] and were estimated using the maximum likelihood estimation method. Statistical significance was set to *p* values < 0.05.

## Results

### Descriptive analysis

Table 1 provides the descriptive statistics of person- and day-level variables. As for the outcome variable, the average fatigue was 2.72 (*SD* = 1.12, ICC = 0.46). The within-person range of fatigue was *M* = 0.55 (*SD* = 0.50): Compared with typical days, the participants reported 2.57 units higher fatigue on several days; they reported 2.14 units lower fatigue on other days. Regarding sleep behavior, time in bed reached 8.0 h on average (*SD* = 1.2, ICC = 0.49). The within-person range of time in bed was *M* = 0.4 h (*SD* = 0.5): Compared with usual days, on a few days, the participants slept 3.5 h less; on other days, they slept 4.4 h longer. Sleep satisfaction at sample mean was 4.0 (*SD* = 1.1, ICC = 0.63). The within-person range of sleep satisfaction was *M* = 0.5 (*SD* = 0.6). As such, compared with typical days, the participants reported 3.0 units lower sleep satisfaction on several days; on other days, they rated 2.1 units higher sleep satisfaction.

**Table 1:** Day- and person-level characteristics

	*M*(*SD*) or %	Range	ICC
Person-level (*N*_person_ = 56)			
Age (years)	83.23 (1.06)	82-86	
Gender (% female)	37.5		
Living arrangements (% living alone)	30.4		
Physical health	4.70 (0.54)	3-5	
Mental health	18.21 (4.29)	9-25	
Day-level (*N*_days_ = 392)			
Time in bed (hours)	8.03 (1.21)	3.67-13.00	0.49
Sleep satisfaction	3.95 (1.05)	1-5	0.63
Positive emotions	3.05 (0.89)	1-5.63	0.37
Negative emotions	1.94 (0.66)	1-4.38	0.68
Fatigue	2.72 (1.12)	1-6	0.46
Number of observations after waking	6.88 (0.68)	2-7	
Number of observations before going to bed	6.98 (0.13)	6-7	

*Note*. ICC = intraclass correlation coefficient

Higher values indicate better health status and sleep satisfaction, feeling more emotions and fatigue during the day

### Within- and between-person associations between sleep and fatigue

**Fig. 1:**
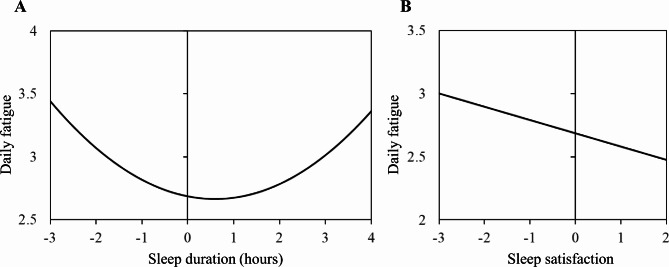
Within-person association between sleep and fatigue. Panel A shows that, on days following short and long time in bed, they felt more fatigue compared with days following average time in bed. Panel B demonstrates that, on days following low levels of satisfaction with sleep, they also felt more fatigue compared with days following average sleep satisfaction

Table 2 presents the results of the multilevel model for the association between sleep and fatigue. At the within-person level, prior-night time in bed was nonlinearly associated with next-day fatigue (γ_50_ = 0.06; Panel A in Fig. 1). Specifically, individuals felt more fatigue on days following short and long time in bed compared with days following average time in bed. Furthermore, satisfaction with prior-night sleep was significantly associated with next-day fatigue (γ_60_ = − 0.11; Panel B in Fig. 1). Specifically, individuals felt more fatigue on days following low levels of satisfaction with sleep compared with days following average sleep satisfaction. In terms of the covariates, within-person coupling between same-day negative emotions and fatigue was observed (γ_30_ = 0.47). In other words, individuals felt more fatigue on days when the participants perceived high levels of negative emotions compared with days with average levels of negative emotions. Time in study and the within-person component of positive emotions were not significantly associated with fatigue.

**Table 2:** Within- and between-person effects of prior-night sleep on next-day fatigue

	*Estimate* (*SE*)
*Fixed effects*	
Level 1 (Within-person effects)	
Intercept (mean), γ_00_	2.69 (0.09)***
Time in study (day), γ_10_	0.00 (0.02)
Same-day positive emotions, γ_20_	–0.11 (0.08)
Same-day negative emotions, γ_30_	0.47 (0.09)***
Prior-night time in bed, γ_40_	–0.07 (0.06)
Prior-night time in bed^2^, γ_50_	0.06 (0.03)*
Prior-night sleep quality, γ_60_	–0.11 (0.05)*
Level 2 (Between-person effects)	
Age, γ_01_	–0.02 (0.10)
Gender, γ_02_	0.06 (0.24)
Living arrangements, γ_03_	–0.27 (0.24)
Physical health, γ_04_	0.30 (0.19)
Mental health, γ_05_	–0.02 (0.03)
Average positive emotions, γ_06_	0.01 (0.14)
Average negative emotions, γ_07_	0.70 (0.22)**
Average time in bed, γ_08_	0.02 (0.10)
Average sleep satisfaction, γ_09_	–0.20 (0.15)
*Random effects*	
Level 1	
Residual	0.60 (0.06)***
Autocorrelation	0.18 (0.07)*
Level 2	
Intercept	0.37 (0.10)***

*Note*. *N* = 56. Unstandardized estimates are presented with standard errors in parentheses. Time in study was centered at the median during the observation period (i.e., seven days), and all between-person variables were centered at sample means

* p < .05. ** *p* < .01. *** *p* < .001

At the between-person level, sleep was not significantly related to fatigue. For the covariates, negative emotions and fatigue were positively correlated (γ_07_ = 0.70), which indicated that individuals were likely to feel more fatigue due to more negative emotions during the observation period compared with typical individuals.

### Follow-up analyses

We explored the reversed associations to examine whether prior-day fatigue was associated with next-night sleep. As summarized in Supplementary Table 1, fatigue was not statistically significantly associated with sleep duration and sleep satisfaction at the between-person and within-person levels. When further examining fatigue-squared interactions with sleep at the within-person levels, the associations between fatigue and sleep remained unchanged across models.

## Discussion

Previous research has indicated that sleep problems (e.g., short sleep duration and low sleep quality) can increase fatigue in middle-aged adults [22, 23]. However, the day-to-day relationship between sleep and fatigue has been less studied in older adults. Using the daily diary method, the present study demonstrated that the participants felt higher levels of next-day fatigue on days with short and long time in bed compared with usual days, even after controlling for relevant covariates. These results supported our hypothesis that individuals would feel high levels of fatigue on days following short and long sleep duration. Additionally, the current and previous studies [22, 23] consistently demonstrated that individuals perceived higher levels of next-day fatigue on days following low levels of sleep satisfaction than usual days, which suggests that the quantity and quality of sleep are independently associated with fatigue.

Although we found that prior-night sleep with a long duration could increase next-day fatigue in older adults, the underlying mechanisms remain unclear. Several explanations are possible for the within-person association between long time in bed and fatigue. The first explanation is the biological mechanism of inflammatory mediators. Although the causal associations are unclear, previous studies suggest that inflammation is associated with neurovegetative symptoms, including sleep and fatigue [13, 53]. Furthermore, Irwin and colleagues [54] conducted a meta-analysis on the relationship between sleep and inflammation and reported that the levels of inflammation indices, such as C-reactive proteins and interleukin-6, were higher in people with long sleep duration than in those with short sleep duration. To date, however, the majority of existing findings were obtained from studies that examined between-person association. Thus, future studies should directly test the biological mechanism in which inflammation mediates the within-person association between long sleep and fatigue.

The second explanation is that prolonged time in bed due to sleep disturbances could cause next-day fatigue. Sleep disturbances can be characterized by multiple symptoms, such as increased sleep onset latency, impaired non-REM sleep, interrupted sleep, and awakening too early in the morning. Indeed, Alfini and colleagues [26] examined the between-person association between objectively measured sleep duration and fatigue and found that people with prolonged awake time after sleep onset reported more fatigue. In contrast, the present study operationalized sleep duration as the time spent in bed from bedtime to waking time, following earlier studies [35, 36]. Thus, we did not assess various aspects of sleep behavior, such as the time awake until and after sleep onset. Future research should objectively measure sleep duration and test whether long sleep duration and fatigue are associated at the within-person level.

Finally, the third explanation is that pathological conditions might be potentially associated with abnormal sleep duration and fatigue. Previous studies [12, 18, 19] suggested that comorbidities and medications were associated with sleep and fatigue at the between-person level. Our participants were relatively healthy and we controlled for physical and mental health, but such pathological mechanisms might underlie the day-to-day relationship between sleep and fatigue.

### Strengths and limitations

The strength of this study is that our study sample was older adults (i.e., 80 years and above). Some studies examining the within-person association between sleep and fatigue incorporated middle-aged adults [22, 23], whereas another study recruited a lifespan sample but included only a limited number of older adults aged 70 years and above [28]. Thus, this study could extend the prior evidence to old age.

However, this study also has several limitations. First, our participants were the so-called old-old. Further examinations are required to test the generalizability of the findings to the general older population by incorporating individuals from the young-old to the oldest-old.

Second, our sample size was relatively small (i.e., *N*_person_ = 56). Although the number exceeded the minimum in estimating multilevel models [55], we might lack statistical power to detect interindividual differences in fatigue. Future research should determine an adequate sample size based on power analysis.

Third, the daily diary method may minimize recall bias but not eliminate it. Although we incorporated relatively healthy older adults, our participants were very old and might have subtle cognitive decline. Thus, the participants could over- or under-estimate self-reports.

Fourth, the measurement of the primary variable should be refined. In this study, because the within-person level reliability of the four-item fatigue subscale of the PANAS-X was low (see the Outcome variable subsection for details), we had to choose one item, “tired.” Regarding sleep behavior, earlier studies examining between-person associations have commonly used the PSQI [24]. However, existing standard measurements of fatigue and sleep were developed based on studies examining interindividual differences. The measures of fatigue and sleep differed across studies [22, 23], which made it difficult to compare results across studies. Further examinations are required to establish measures capturing within-person processes.

Lastly, there could be unmeasured covariates. For instance, the previous and current studies [22, 23] did not assess daytime activities in detail. Cho and colleagues [56] reported that loneliness was associated with sleep problems and fatigue in older adults. Thus, social mechanisms, such as the lack of interaction with others during the daytime, may lead to long sleep duration and fatigue.

## Conclusions

Using the daily diary method, this study found that sleep behavior and fatigue fluctuated on a daily basis and are associated at the within-person level in older adults. Specifically, individuals were more likely to experience fatigue on days following short and long time in bed and days following low levels of sleep satisfaction compared with typical days. Considering that sleep is a modifiable health behavior, appropriate management of sleep behavior may reduce fatigue. Interventions aimed at managing both sleep duration and sleep quality should be developed to mitigate fatigue in older adults. Such interventions may, in turn, play a pivotal role in the prevention of frailty.

The majority of studies compared people with and without sleep problems and examined the between-person association between sleep and fatigue. In contrast, recent studies have begun comparing days when people sleep well and those when they do not and examine the within-person association. Further research should investigate between- and within-person associations to better understand daily behaviors, including sleep and fatigue.

### Electronic supplementary material

Below is the link to the electronic supplementary material.

Supplementary Material 1

## Data Availability

Datasets analyzed during this study are not allowed to be publicly shared due to ethical restrictions but are available from the corresponding author on reasonable request.

## List of abbreviations

ICC: Interclass correlation coefficient
M: Mean
PANAS-X: Expanded version of the Positive Affect-Negative Affect Schedule
PSQI: Pittsburgh Sleep Quality Index
SD: Standard deviation
WHO-5-J: Japanese version of the WHO-Five Well-Being Index

## References

[1] Fried LP, Tangen CM, Walston J, Newman AB, Hirsch C, Gottdiener J (2001). Frailty in older adults: evidence for a phenotype. Journals Gerontol Ser a Biol Sci Med Sci.

[2] Ensrud KE, Ewing SK, Taylor BC, Fink HA, Cawthon PM, Stone KL (2008). Comparison of 2 frailty indexes for prediction of falls, disability, fractures, and death in older women. Arch Intern Med.

[3] Knoop V, Costenoble A, Vella Azzopardi R, Vermeiren S, Debain A, Jansen B (2019). The operationalization of fatigue in frailty scales: a systematic review. Ageing Res Rev.

[4] Hardy SE, Studenski SA (2008). Fatigue predicts mortality in older adults. J Am Geriatr Soc.

[5] Moreh E, Jacobs JM, Stessman J (2010). Fatigue, function, and mortality in older adults. Journals Gerontol Ser a Biol Sci Med Sci.

[6] Hardy SE, Studenski SA (2008). Fatigue and function over 3 years among older adults. Journals Gerontol Ser a Biol Sci Med Sci.

[7] Vestergaard S, Nayfield SG, Patel KV, Eldadah B, Cesari M, Ferrucci L (2009). Fatigue in a representative population of older persons and its association with functional impairment, functional limitation, and disability. Journals Gerontol Ser a Biol Sci Med Sci.

[8] Avlund K (2010). Fatigue in older adults: an early indicator of the aging process?. Aging Clin Exp Res.

[9] Zengarini E, Ruggiero C, Pérez-Zepeda MU, Hoogendijk EO, Vellas B, Mecocci P (2015). Fatigue: relevance and implications in the aging population. Exp Gerontol.

[10] Meng H, Hale L, Friedberg F (2010). Prevalence and predictors of fatigue among middle-aged and older adults: evidence from the Health and Retirement Study. J Am Geriatr Soc.

[11] Santos-Eggimann B, Cuénoud P, Spagnoli J, Junod J (2009). Prevalence of frailty in middle-aged and older community-dwelling europeans living in 10 countries. Journals Gerontol Ser a Biol Sci Med Sci.

[12] Liao S, Ferrell BA (2000). Fatigue in an older population. J Am Geriatr Soc.

[13] Beyer I, Njemini R, Bautmans I, Demanet C, Bergmann P, Mets T (2012). Inflammation-related muscle weakness and fatigue in geriatric patients. Exp Gerontol.

[14] Ancoli-Israel S (2009). Sleep and its disorders in aging populations. Sleep Med.

[15] Loh KP, Zittel J, Kadambi S, Pandya C, Xu H, Flannery M (2018). Elucidating the associations between sleep disturbance and depression, fatigue, and pain in older adults with cancer. J Geriatr Oncol.

[16] Hawker GA, French MR, Waugh EJ, Gignac MAM, Cheung C, Murray BJ (2010). The multidimensionality of sleep quality and its relationship to fatigue in older adults with painful osteoarthritis. Osteoarthr Cartil.

[17] Vitiello MV, McCurry SM, Shortreed SM, Baker LD, Rybarczyk BD, Keefe FJ et al. Short-term improvement in insomnia symptoms predicts long-term improvements in sleep, pain, and fatigue in older adults with comorbid osteoarthritis and insomnia. Pain. 201;155(8):1547–54. 10.1016/j.pain.2014.04.032 PMC4104256 24793909

[18] Goldman SE, Ancoli-Israel S, Boudreau R, Cauley JA, Hall M, Stone KL (2008). Sleep problems and associated daytime fatigue in community-dwelling older individuals. Journals Gerontol Ser a Biol Sci Med Sci.

[19] Endeshaw YW. Do sleep complaints predict persistent fatigue in older adults? J Am Geriatr Soc. 201;63(4):716–21. 10.1111/jgs.13329 PMC4831048 25851828

[20] Molenaar PCM (2004). A manifesto on psychology as idiographic science: bringing the person back into scientific psychology, this time forever. Meas Interdiscip Res Perspect.

[21] Curran PJ, Bauer DJ (2011). The disaggregation of within-person and between-person effects in longitudinal models of change. Annu Rev Psychol.

[22] Harris AL, Carmona NE, Moss TG, Carney CE. Testing the contiguity of the sleep and fatigue relationship: a daily diary study. Sleep. 2020;44(5). 10.1093/sleep/zsaa252 33245330

[23] DePasquale N, Crain T, Buxton OM, Zarit SH, Almeida DM, Pruchno R (2019). Tonight’s sleep predicts tomorrow’s fatigue: a daily diary study of long-term care employees with nonwork caregiving roles. Gerontologist.

[24] Buysse DJ, Reynolds CF, Monk TH, Berman SR, Kupfer DJ (1989). The Pittsburgh sleep quality index: a new instrument for psychiatric practice and research. Psychiatry Res.

[25] Carney CE, Buysse DJ, Ancoli-Israel S, Edinger JD, Krystal AD, Lichstein KL (2012). The consensus sleep diary: standardizing prospective sleep self-monitoring. Sleep.

[26] Alfini AJ, Schrack JA, Urbanek JK, Wanigatunga AA, Wanigatunga SK, Zipunnikov V (2020). Associations of actigraphic sleep parameters with fatigability in older adults. Journals Gerontol Ser a Biol Sci Med Sci.

[27] Glynn NW, Santanasto AJ, Simonsick EM, Boudreau RM, Beach SR, Schulz R (2015). The Pittsburgh Fatigability Scale for older adults: development and validation. J Am Geriatr Soc.

[28] Wrzus C, Wagner GG, Riediger M (2014). Feeling good when sleeping in? Day-to-day associations between sleep duration and affective well-being differ from youth to old age. Emotion.

[29] Gondo Y, Masui Y, Kamide K, Ikebe K, Arai Y, Ishizaki T, Pachana NA (2017). SONIC study: a longitudinal cohort study of the older people as part of a centenarian study. Encyclopedia of Geropsychology.

[30] Arai Y, Zarit SH, Kumamoto K, Takeda A (2003). Are there inequities in the assessment of Dementia under Japan’s LTC insurance system?. Int J Geriatr Psychiatry.

[31] Watson D, Clark L (1999). The PANAS-X: Manual for the positive and negative affect schedule-expanded form.

[32] Nishikawa K (2012). Is surprise a Neutral emotion? Comparative analysis using state and trait versions of PANAS–X. Kandai Psychol Reports.

[33] Hartz A, Bentler S, Watson D (2003). Measuring fatigue severity in primary care patients. J Psychosom Res.

[34] Parrish BP, Zautra AJ, Davis MC (2008). The role of positive and negative interpersonal events on daily fatigue in women with Fibromyalgia, rheumatoid arthritis, and Osteoarthritis. Heal Psychol.

[35] Seegers V, Petit D, Falissard B, Vitaro F, Tremblay RE, Montplaisir J (2011). Short sleep duration and body mass index: a prospective longitudinal study in preadolescence. Am J Epidemiol.

[36] Vollmer C, Jankowski KS, Díaz-Morales JF, Itzek-Greulich H, Wüst-Ackermann P, Randler C (2017). Morningness–eveningness correlates with sleep time, quality, and hygiene in secondary school students: a multilevel analysis. Sleep Med.

[37] Mccrae CS, Mcnamara JPH, Rowe MA, Dzierzewski JM, Dirk J, Marsiske M (2008). Sleep and affect in older adults: using multilevel modeling to examine daily associations. J Sleep Res.

[38] Abraham O, Pu J, Schleiden LJ, Albert SM (2017). Factors contributing to poor satisfaction with sleep and healthcare seeking behavior in older adults. Sleep Heal.

[39] Park S (2014). Associations of physical activity with sleep satisfaction, perceived stress, and problematic internet use in Korean adolescents. BMC Public Health.

[40] Koyano W, Shibata H, Nakazato K, Haga H, Suyama Y (1991). Measurement of competence: reliability and validity of the TMIG Index of competence. Arch Gerontol Geriatr.

[41] Shibata H, Sugisawa H, Watanabe S (2001). Functional capacity in elderly Japanese living in the community. Geriatr Gerontol Int.

[42] Fujiwara Y, Yoshida H, Amano H, Fukaya T, Liang J, Uchida H (2008). Predictors of improvement or decline in instrumental activities of daily living among community-dwelling older Japanese. Gerontology.

[43] Shimada H, Sawyer P, Harada K, Kaneya S, Nihei K, Asakawa Y (2010). Predictive validity of the classification schema for functional mobility tests in instrumental activities of daily living decline among older adults. Arch Phys Med Rehabil.

[44] Tanimoto Y, Watanabe M, Sun W, Hirota C, Sugiura Y, Kono R (2012). Association between muscle mass and disability in performing instrumental activities of daily living (IADL) in community-dwelling elderly in Japan. Arch Gerontol Geriatr.

[45] Awata S, Bech, Yoshida S, Hirai M, Suzuki S, Yamashita M (2007). Reliability and validity of the Japanese version of the World Health Organization-Five Well-Being Index in the context of detecting depression in diabetic patients. Psychiatry Clin Neurosci.

[46] Awata S, Bech P, Koizumi Y, Seki T, Kuriyama S, Hozawa A (2007). Validity and utility of the Japanese version of the WHO-Five Well-Being Index in the context of detecting suicidal ideation in elderly community residents. Int Psychogeriatr.

[47] Russell C, Wearden AJ, Fairclough G, Emsley RA, Kyle SD (2016). Subjective but not actigraphy-defined sleep predicts next-day fatigue in Chronic Fatigue Syndrome: a prospective daily diary study. Sleep.

[48] Sin NL, Almeida DM, Crain TL, Kossek EE, Berkman LF, Buxton OM (2017). Bidirectional, temporal associations of sleep with positive events, affect, and stressors in daily life across a week. Ann Behav Med.

[49] Sato A, Yasuda A (2001). Development of the Japanese version of positive and negative affect schedule (PANAS) scales. Japanese J Personal.

[50] Watson D, Clark LA, Tellegen A (1988). Development and validation of brief measures of positive and negative affect: the PANAS scales. J Pers Soc Psychol.

[51] Bolger N, Laurenceau J (2013). Intensive longitudinal methods: an introduction to diary and experience sampling research.

[52] Little RJA, Rubin DB (2002). Statistical analysis with missing data.

[53] Majd M, Saunders EFH, Engeland CG (2020). Inflammation and the dimensions of depression: a review. Front Neuroendocrinol.

[54] Irwin MR, Olmstead R, Carroll JE (2016). Sleep disturbance, sleep duration, and inflammation: a systematic review and meta-analysis of cohort studies and experimental sleep deprivation. Biol Psychiatry.

[55] McNeish DM, Stapleton LM (2016). The effect of small sample size on two-level model estimates: a review and illustration. Educ Psychol Rev.

[56] Cho JHJ, Olmstead R, Choi H, Carrillo C, Seeman TE, Irwin MR (2019). Associations of objective versus subjective social isolation with sleep disturbance, depression, and fatigue in community-dwelling older adults. Aging Ment Heal.

