# Supplementary material for: Association between prior-night sleep and next-day fatigue in older adults: a daily diary study
Source: BMC Geriatr. 2023 Dec 7;23:817. doi: 10.1186/s12877-023-04539-0 (PMC10704841; doi:10.1186/s12877-023-04539-0)
Supplement: Supplementary file 1 — Supplementary Material 1 [file 12877_2023_4539_MOESM1_ESM.docx]

**Supplementary Table 1**

Within- and between-person effects of prior-day fatigue on next-night sleep

|  | Sleep duration |  | Sleep satisfaction |
| --- | --- | --- | --- |
|  | *Estimate* (*SE*) |  | *Estimate* (*SE*) |
| *Fixed effects* |  |  |  |
| Level 1 (Within-person effects) |  |  |  |
| Intercept (mean), γ_00_ | 8.02 (0.13)*** |  | 3.95 (0.09)*** |
| Time in study (day), γ_10_ | 0.02 (0.03) |  | —0.05 (0.03) |
| Prior-day positive emotions, γ_20_ | 0.05 (0.10) |  | —0.13 (0.11) |
| Prior-day negative emotions, γ_30_ | 0.02 (0.10) |  | —0.19 (0.03) |
| Prior-day fatigue, γ_40_ | 0.16 (0.10) |  | 0.14 (0.11) |
| Level 2 (Between-person effects) |  |  |  |
| Age, γ_01_ | —0.14 (0.14) |  | 0.03 (0.09) |
| Gender, γ_02_ | —0.26 (0.33) |  | —0.19 (0.22) |
| Living arrangements, γ_03_ | —0.13 (0.34) |  | 0.09 (0.22) |
| Physical health, γ_04_ | 0.01 (0.27) |  | 0.07 (0.18) |
| Mental health, γ_05_ | 0.01 (0.04) |  | 0.07 (0.03)** |
| Average positive emotions, γ_06_ | 0.02 (0.20) |  | —0.25 (0.13) |
| Average negative emotions, γ_07_ | 0.49 (0.36) |  | 0.07 (0.23) |
| Average fatigue, γ_08_ | —0.13 (0.34) |  | —0.32 (0.18) |
| *Random effects* |  |  |  |
| Level 1 |  |  |  |
| Residual | 0.57 (0.05)*** |  | 0.68 (0.06)*** |
| Autocorrelation | 0.05 (0.08) |  | 0.04 (0.08) |
| Level 2 |  |  |  |
| Intercept | 0.86 (0.18)*** |  | 0.30 (0.08)*** |

*Note*. *N* = 56. Unstandardized estimates are presented with standard errors in parentheses. Sleep duration is measured in hours, and sleep satisfaction ranges from 1 to 5 (not satisfied to satisfied). Time in study was centered at the median during the observation period (i.e., seven days), and all between-person variables were centered at sample means.

** p* < .05. ** *p* < .01. *** *p* < .001.
